# Expression of C-X-C chemokine receptor types 1/2 in patients with gastric carcinoma: Clinicopathological correlations and significance

**DOI:** 10.3892/ol.2012.1043

**Published:** 2012-11-23

**Authors:** JUN PU WANG, WAN MING HU, KUAN SONG WANG, JUN YU, BAI HUA LUO, CHANG WU, ZHI HONG CHEN, GENG QIU LUO, YU WU LIU, QIN LAI LIU, YAN XIAO, HAI YAN ZHOU, XIAO JING YANG, HAI YING JIANG, JING HE LI, JI FANG WEN

**Affiliations:** 1 Department of Pathology, School Of Basic Medicine; Central South University, Hunan, Changsha 410013, P.R. China; 2 Third Xiang-ya Hospital, Central South University, Hunan, Changsha 410013, P.R. China

**Keywords:** chemokine, C-X-C chemokine receptor types 1/2, gastric carcinoma, tumor

## Abstract

C-X-C chemokine receptor types 1/2 (CXCR1/2) may play multiple roles in the development and progression of a number of types of tumor. The abnormal expression of CXCR1/2 in various types of malignant tumors has been reported, but less is known with regard to gastric carcinoma. The present study was preliminarily conducted to elucidate the correlation between clinicopathological factors and the immunohistochemical expression of CXCR1/2 in patients with gastric carcinoma. The expression of CXCR1/2 in 69 specimens of sporadic gastric carcinoma and their corresponding non-neoplastic mucosa obtained by gastrectomy was assayed by immunohistochemistry (IHC) using a polyclonal anti-CXCR1/2 antibody. ERK1/2 and AKT phosphorylation and the expression of indicators of proliferation, growth and apoptosis (Bcl-2 and Bax, Cyclin D1, EGFR and Ki-67), angiogenesis (VEGF and CD34), invasion and metastasis (MMP-9, MMP-2, TIMP-2 and E-cadherin) were also detected by IHC. A total of 68 (98.6%) of the 69 patients with gastric carcinoma were found to have positive CXCR1/2 expression, which appeared to be significantly higher in gastric carcinoma compared with corresponding non-neoplastic mucosa tissues. The expression of CXCR1/2 in gastric carcinoma was significantly associated with invasion, metastasis and TNM staging (P<0.001). Correlation analysis between CXCR1/2 and pAKT (P=0.032), pERK (P<0.001), Cyclin D1 (P=0.049), EGFR (P=0.013), Bcl-2 (P=0.003), microvessel density (P=0.001), MMP-9 (P=0.013) and MMP-2 (P=0.027) expression using the Spearman test showed significant correlation in gastric carcinoma. Univariate and multivariate logistic regression analysis showed that, compared with negative or weak expression, overexpression of CXCR1/2 protein was a significant risk factor for TNM stage (P<0.001). These results preliminarily suggest that CXCR1/2 may be a useful maker for progression of the tumors and a promising target for gastric carcinoma therapy.

## Introduction

Gastric carcinoma is one of the most deadly types of cancer worldwide (1,2), especially in China (3). Despite making advances in treatment and putting effort into research over the past few decades, the outcome of gastric cancer remains unsatisfactory, and the overall 5-year survival rate of advanced gastric adenocarcinoma patients is low. Therefore, improvement in the therapy of gastric cancer now depends on improving our understanding of the complex molecular mechanisms governing the progression and aggressiveness of the disease. Uncontrolled proliferation, invasion and metastasis as a whole is a major poor prognostic factor for advanced gastric cancer (4). It is well known that there is a close correlation between inflammation and cancer; early in 1863, Virchow hypothesized that the origin of cancer was at sites of chronic inflammation (5). Non-resolving inflammation plays a critical role in the development and progression of gastric cancer (6,7), including in the dialectical correlation between inflammation and tumor progression, chemokine receptors and their ligands, an important class of non-resolving inflammatory factors, are involved in carcinogenic, proliferative, growth, invasive, metastatic and drug resistance processes (8–11).

C-X-C chemokine receptor types 1/2 (CXCR1/2) belong to the chemokine receptor family, which consists of G protein-coupled receptors containing 7 transmembrane domains. CXCR1/2 are receptors for interleukin-8 (IL-8) and transduce the signal through a G protein-activating second messenger system. CXCR1 and CXCR2 proteins have a single poly-peptide chain which is 350 and 355 or 360 amino acids in length, respectively, which share 76% amino acid identity to one another with the highest homology over the membrane-spanning regions and significant divergence at both N- and C-termini (12,13). CXCR1/2 are expressed mainly on neutrophils and were originally characterized by their ability to induce the chemotaxis of leukocytes. Recently, it was found that CXCR1/2 are overexpressed in numerous solid tumors, and the studies revealed a close correlation with proliferation, angiogenesis, invasion, metastasis and drug resistance of the tumor (14–19). Although there have been some studies on CXCR1/2 in several cancer types and there have been a few reports on the role of CXCR1/2 in gastric carcinoma (20), to date, the significance of CXCR1/2 expression in gastric cancer progression has not been evaluated in detail.

To determine the functional role of CXCR1/2 in the progression of gastric carcinoma, based on the literature review and our previous study (21), in the present study, we investigated CXCR1/2 expression in tumors of patients diagnosed with primary gastric carcinoma and in corresponding non-neoplastic mucosa. We preliminarily discuss the correlation between the immunohistochemical expression of CXCR1/2 and clinicopathological features, phosphorylation of ERK1/2 and AKT and the expression of relevant indicators of proliferation, growth and apoptosis (Bcl-2, Bax, Cyclin D1, EGFR and Ki-67), angiogenesis (VEGF and CD34), invasion and metastasis (MMP-9, MMP-2, TIMP-2 and E-cadherin).

## Materials and methods

### Patients and specimens

This study was conducted on 69 primary and sporadic gastric adenocarcinoma tissue samples and their corresponding non-neoplastic mucosa specimens retrieved from the archives at the Department of Pathology of Xiang-ya Hospital of Central South University (Changsha, China) between 2008 and 2010. The protocol followed the ethical guidelines of the Declaration of Helsinki, and informed consent was obtained from all patients before the study. No patients had received chemotherapy or radiotherapy prior to surgery. Tissue blocks of non-neoplastic mucosa (>5 cm away from the edge of tumor) were obtained. The clinicopathological findings were determined according to the AJCC tumor-node-metastasis (TNM) staging system (22). The patients' data and histopathological characteristics of the tumors are summarized in Table I.

### Antibodies

Phosphorylated antibodies (pAKT-Ser^473^, anti-AKT, pERK-Thr^202^/Tyr^204^ and anti-ERK) were obtained from Anbo Biotechnology Co., Ltd. (San Francisco, CA, USA) and the following antibodies were purchased from Santa Cruz Biotechnology, Inc. (Santa Cruz, CA, USA): CXCR1/2, Bcl-2, Bax, Cyclin D1, EGFR, Ki-67, VEGF, CD34, MMP-9, MMP-2, TIMP-2 and E-cadherin. The StreptAvidin Biotin Complex (SABC) kit (Wuhan Boster Bio-Engineering Ltd. Co., China) was used according to the manufacturer's instructions.

### Immunohistochemical analysis

Immunohistochemistry (IHC) was performed as previously described (21). More than 10 serial thin (4 *μ*m) sections were cut from each paraffin block. The sections were deparaffinized and endogenous peroxidase activity was blocked. The sections were then pre-treated in antigen retrieval buffer (citrate buffer, pH 6.0, at 100°C, 2 min in a pressure cooker) and stained with primary antibodies CXCR1/2, pAKT-Ser^473^, AKT, pERK-Thr^202^/Tyr^204^, ERK, Bcl-2, Bax, Cyclin D1, EGFR, Ki-67, VEGF, CD34, MMP-9, MMP-2, TIMP-2 and E-cadherin (diluted 1:200). IgG2b-stained sections were used as negative controls. Slides were then washed and incubated for 1 h with the appropriate horseradish peroxidase-conjugated secondary antibody. Diaminobenzidine (DAB) was used as the chromogen and sections were counterstained with hematoxylin.

### Clinicopathological and immunohistochemical assessment

The tumors were staged by two observers who had no prior knowledge of the results of the assays, according to the 7th edition of the AJCC tumor-node-metastasis (TNM) classifications. The immunohistochemical expression of the indicators was independently assessed by two pathologists, without knowledge of the clinical data. The distribution of the immunohistochemical expression of the indicators was semi-quantitatively assessed by estimating the proportion and intensity of positively stained tumor cells. According to previous studies (23,24), in brief, the adjusted Allred scoring system was applied to evaluate each entire slide using light microscopy. First, the proportion score (PS) was assigned using a 0-to-4 scale: 0 for 0–5% positive tumor cells, 1 for 6–25% positive tumor cells, 2 for 26–50% positive tumor cells, 3 for 51–75% positive tumor cells and 4 for >75% positive tumor cells. The intensity score (IS) was based on a 4-point system: 0, 1, 2 and 3 (for no, light, medium or dark staining, respectively). The proportion and intensity scores were added to obtain a total score. When a total score was 0 or 1, the intensity of immunostaining in the tissue was considered negative; the intensity was weak when a total score was between 2 and 4; and the intensity was strong when a total score was ≥5. Clinicopathological factors, including age, gender, staging, ERK1/2 and AKT phosphorylation and the expression of indicators of proliferation, growth and apoptosis (Bcl-2, Bax, Cyclin D1, EGFR and Ki-67), angiogenesis (VEGF and CD34), invasion and metastasis (MMP-9, MMP-2, TIMP-2 and E-cadherin) were analyzed for an association with CXCR1/2 expression.

### Statistical analysis

The SPSS 13.0 software system (SPSS, Inc., Chicago, IL, USA) was used for statistical analysis. The Spearman correlation was used, when appropriate, to analyze the significance of the correlation between CXCR1 protein expression and tumor characteristics, including age, gender, staging, ERK1/2 and AKT phosphorylation and expression of indicators of proliferation, growth and apoptosis, invasion and metastasis. Uni- and multivariate logistic regression analysis was performed to determine factors associated with tumor stage. P<0.05 was considered to indicate a statistically significant result.

## Results

### Association between CXCR1/2 expression and clinicopathological factors of gastric carcinoma

**Figure 1.**
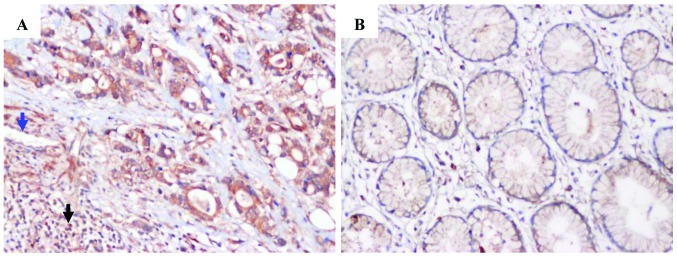
Representative immunohistochemical staining for CXCR1/2 in corresponding non-neoplastic mucosa tissue and tumor. (A) Tumor tissue with strong expression. CXCR1/2 were also present in some leukocytes (black arrowhead) and vascular endothelial cells (blue arrowhead). (B) Corresponding non-neoplastic mucosa tissue. Original magnification, x200. IgG stainingwas used as a negative control. CXCR1/2, C-X-C chemokine receptor types 1/2.

Positive staining for CXCR1/2 was shown in 68 (98.6%) of the 69 tumor specimens (Table II). CXCR1/2 showed membrane and cytoplasmic expression in tumor cells and also in some leukocytes and vascular endothelial cells (Fig. 1). Based on CXCR1/2 expression levels, demographic characteristics and tumor status were analyzed (Table II). Table II shows that as CXCR1/2 expression increased in the tumor, so did the overall tumor stage. Of 68 tumors with positive CXCR1/2 expression, 60 (88.2%) cases were stage II, III and IV, but only 8 (11.8%) were stage I. According to the evaluation of the CXCR1/2 immnunostaining, CXCR1/2 expression was significantly correlated with TNM stage, T stage and N stage (P<0.001). However, no correlation was observed between CXCR1/2 expression and gender, age and tumor differentiation.

### Association between expression of CXCR1/2 and indicators of phosphorylation, proliferation, growth, apoptosis, angiogenesis, invasion and metastasis

**Figure 2.**
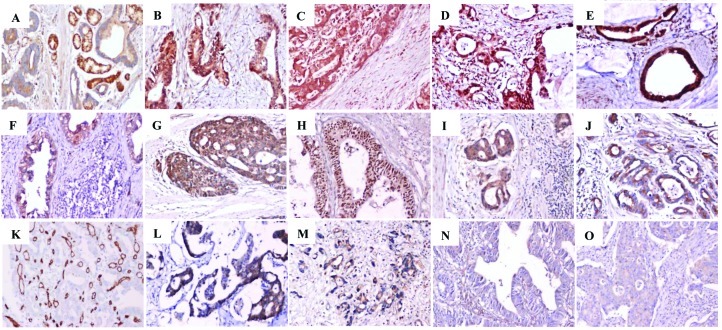
In gastric carcinoma, representative immunostaining for phosphorylation indicators: (A) AKT, (B) pAKT, (C) ERK, (D) pERK; proliferation, growth and apoptosis indicators: (E) Bcl-2, (F) Bax, (G) Ki-67, (H) Cyclin D1, (I) EGFR; angiogenesis indicators: (J) VEGF, (K) CD34 to calculate microvessel density; invasion and metastasis indicators: (L) MMP-2, (M) MMP-9, (N) TIMP-2, (O) E-cadherin. Original magnification, x200. IgG staining was used as a negative control.

Correlation analysis between the expression of CXCR1/2 and the indicators of phosphorylation (AKT, ERK, pAKT and pERK), proliferation, growth and apoptosis (Bcl-2, Bax, Cyclin D1, EGFR and Ki-67), angiogenesis (VEGF and CD34), invasion and metastasis (MMP-9, MMP-2, TIMP-2 and E-cadherin) using the Spearman correlation test revealed that CXCR1/2 expression was significantly correlated with pAKT, pERK, Cyclin D1, EGFR, Bcl-2, microvessel density (MVD), MMP-9 and MMP-2 (P=0.032, P<0.001, P=0.049, P=0.013, P=0.003, P=0.001, P=0.013 and P=0.027, respectively), but CXCR1/2 and AKT, ERK, Ki-67, Bax, VEGF, TIMP-2 and E-cadherin expression were not significantly correlated in gastric carcinoma (Fig. 2 and Table III).

### Association between expression of pAKT and pERK and indicators of proliferation, growth, apoptosis, angiogenesis, invasion and metastasis

Positive immunohistochemical reaction for AKT, pAKT, ERK and pERK in tumor cells was characterized by positive staining in the membrane and cytoplasm (Fig. 2A–D). Based on the immnunostaining evaluation, the expression of pAKT was significantly correlated with Ki-67, EGFR, Bcl-2, VEGF and MMP-2 expression (P=0.001, P=0.029, P<0.001, P=0.003 and P=0.041, respectively), but not with Cyclin D1, Bax, MVD, MMP-9, TIMP-2 and E-cadherin expression. pERK expression was significantly correlated with Ki-67, EGFR, Bcl-2, MMP-9 and MMP-2 expression (P=0.013, P=0.002, P<0.001, P=0.003 and P=0.010, respectively), and tended to correlate with Cyclin D1, Bax, MVD and TIMP-2 expression (P=0.098, P=0.081, P=0.073 and P=0.084, respectively), but not with VEGF and E-cadherin expression (Table IV).

### Factors associated with tumor stage

Based on a univariate analysis, CXCR1/2, pERK and MVD expression were significantly associated with high TNM stage, and the odds ratios (ORs) were 39.291, 5.186 and 13.383, respectively, which suggested that cases with strong CXCR1 expression, strong ERK phosphorylation and high MVD had a 39.291-, 5.186- and 13.383-fold higher risk for high TNM stage, compared with negative and weak CXCR1 expression and ERK phosphorylation and low MVD, respectively. The other detected indicators were not significantly associated with high TNM stage. The multivariate analysis indicated that only CXCR1/2 and MVD expression were significantly associated with high TNM stage, with ORs of 204.793 and 28.905, respectively (Table V).

A univariate analysis showed that poor tumor differentiation, strong CXCR1/2 expression and high MVD were significant risk factors for T stage, with ORs of 3.534 (1/0.283), 4.039 and 3.855, respectively. Tumor differentiation, CXCR1/2, pAKT, Ki-67 and EGFR expression were associated with high T stage by the multivariate analysis (Table VI). A univariate analysis indicated that CXCR1/2, pERK, EGFR and MVD expression were significant risk factors for T stage; and the significance of CXCR1/2, pERK, EGFR, VEGF and MVD expression was preserved using multivariate analysis (Table VII). Taken together, strong CXCR1/2 expression is a significant risk factor for T stage, N stage and TNM stage in gastric carcinoma.

## Discussion

Previous studies have shown that cancer cells from numerous types of cancer express higher levels of the chemokine receptors (25–27). Chemokine receptors and their ligands were believed to be involved in all stages of certain types of cancer, including influencing the tumor microenvironment (28,29), malignant cell survival and growth (30), angiogenesis (28), invasion (31) and metastasis (10,11,32). A greater understanding of the chemokine receptor system in malignancy would not only add to our knowledge of the pathogenesis of cancer, but may also suggest new treatment targets for development. CXCR1/2, members of the chemokine receptor family, have been studied in several types of cancer, showing a close correlation with drug resistance, survival, growth, angiogenesis, invasion and metastasis in breast cancer (16), melanoma (17), pancreatic cancer (19,33) and colon cancer (18). In melanoma, knockdown of the receptors or the use of antagonists or neutralizing antibodies against CXCR1/2 affected cell proliferation, migration and tumor growth, strongly indicating the involvement of these receptors in melanoma progression (34).

In gastric carcinoma there is little information concerning the expression of CXCR1/2 proteins, which are generally believed to play a role in tumor progression by interacting with their ligands. In the present study, we examined CXCR1/2 protein expression in gastric carcinoma, and CXCR1/2 expression was immunohistochemically detected in 68 (98.6%) of the 69 tumor specimens. The expression level of CXCR1/2 was higher in gastric carcinoma than in the corresponding non-neoplastic mucosa in certain cases.

Previous studies have indicated that CXCR1/2 expression is significantly correlated with invasion, metastasis and advanced TNM stage in patients with malignant melanoma (35) and prostate cancer (36). Our current data show that the membrane and cytoplasmic expression of CXCR1/2 in gastric carcinoma cells was positively correlated with advanced T stage, N stage and overall TNM stage. T stage and N stage represent invasion and metastasis degree, respectively (22). These data indicate that CXCR1/2 may be involved in the invasion and metastasis of gastric carcinoma. Univariate and multivariate analysis revealed that strong CXCR1/2 expression was a significant risk factor for T stage, N stage and TNM stage, from which CXCR1/2 expression appears to play an overlooked role in the development and progression of gastric carcinoma, as does MVD. Our findings further support the hypothesis that there is an association between CXCR1/2 expression and cancer cell invasion and metastasis in certain cancer types (14,16,37,38).

Several studies have indicated that chemokine receptors play multiple roles in the development and progression of a number of tumors via various mechanisms (14,39–42). To investigate the possible mechanisms of CXCR1/2 involvement in the progression of gastric carcinoma, we examined the immunohistochemical expression of ERK1/2 and AKT phosphorylation and the expression of relevant indicators of proliferation, growth and apoptosis (Bcl-2 and Bax, Cyclin D1, EGFR and Ki-67), angiogenesis (VEGF and CD34), invasion and metastasis (MMP-9, MMP-2, TIMP-2 and E-cadherin) in primary gastric carcinoma and its corresponding nonneoplastic mucosa, which were involved in the regulation of tumor proliferation, growth, angiogenesis, invasion and metastasis. Evaluation of the correlation between the expression of CXCR1/2 and the indicators using the Spearman correlation analysis revealed that CXCR1/2 expression was positively correlated with Cyclin D1, EGFR, Bcl-2, MVD, MMP-9 and MMP-2. CD34 is a marker of MVD and reflects angiogenesis, which plays an important role in the growth and invasion of tumors (43). The cell cycle regulatory protein Cyclin D1 may contribute to TNM classification, histological differentiation, perineural invasion, DNA ploidy, S-phase fraction, expression of Ki-67 and mitotic index. EGFR has been shown to be associated with tumor proliferation and growth, Bcl-2 inhibits tumor apoptosis and MMP-9 and MMP-2 play an indispensable role in tumor invasion and metastasis (44,45). Based on our results, it is possible that via the upregulation of Cyclin D1, EGFR, Bcl-2, MMP-9 and MMP-2 expression and MVD, CXCR1/2 and their ligands are involved in mediating proliferation, growth, angiogenesis, invasion and metastasis of gastric carcinoma.

**Figure 3.**
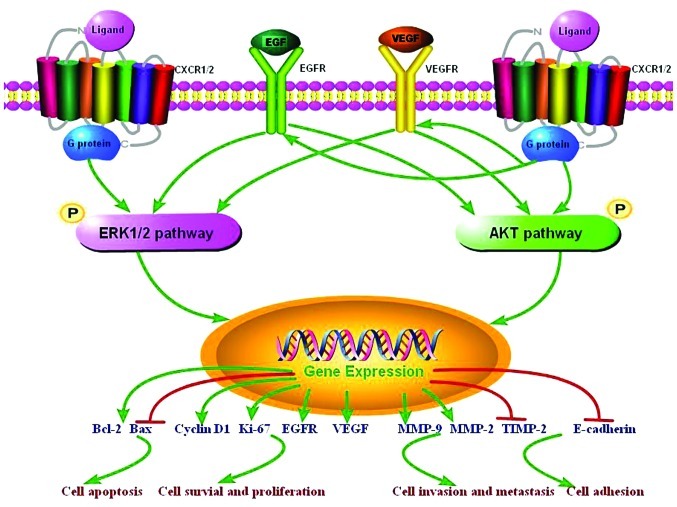
CXCR1/2 receptor/ligand signaling pathways in gastric carcinoma. CXCR1/2, C-X-C chemokine receptor types 1/2.

Other studies (14,15) have found that abnormal phosphorylation of ERK1/2 and AKT was closely associated with proliferation, growth, angiogenesis, invasion and metastasis of tumors, and played as an intermediary between CXCR1/2 and their downstream molecular indicators. Following stimulation of CXCR1/2 receptors with the ligands, for example IL-8, heterotrimeric small G proteins are activated and promote the activation of the primary effector, such as phosphatidyl-inositol-3-kinase, one of the principal targets of the CXCR1/2 subunits. Activation of the phosphatidyl-inositol-3-kinase may result in increased phosphorylation of its substrate serine/threonine kinase, PKB/AKT (46), promoting the activation of AKT or MAPK signaling cascades. These signaling pathways have been shown to promote protein translation and regulate the activity of a range of transcription factors, and are likely to induce the transcription of multiple genes involved in angiogenesis, cell cycle regulation, migration, invasion and the evasion of apoptosis (14). Increased AKT expression and activity by CXCR1/2 receptor/ligand signaling have been detected in multiple forms of cancer, which is consistent with poor tumor progression (47). A study conducted by one group suggests that CXCR1/2 receptor/ligand signaling not only induces activation of AKT but also increases the expression of AKT in androgen-independent prostate cancer cell lines (48). The CXCR1/2 receptor/ligand pathway also regulates the activity of the activation of MAPK signaling cascade, with downstream phosphorylation of ERK1/2 detected in cancer cells (48–50). Certain studies conducted in ovarian and lung cancer cell lines showed that the CXCR1/2 receptor/ligand pathway transactivates EGFR, promoting the downstream activation of MAPK signaling and mediating cell proliferation and survival (49,50). Our data showed that strong CXCR1/2 expression was positively associated with the phosphorylation of AKT and ERK. Further analysis indicated that the expression of pAKT was significantly correlated with Ki-67, EGFR, Bcl-2, VEGF and MMP-2 expression, and pERK expression was significantly correlated with Ki-67, EGFR, Bcl-2, MMP-9 and MMP-2 expression and tended to correlate with Cyclin D1, Bax, MVD and TIMP-2 expression. Therefore, these results, to a certain extent, suggest that CXCR1/2 receptor/ligand signaling plays a significant role in the progression of gastric carcinoma by means of ERK1/2 and AKT phosphorylation, two important pathways (Fig. 3).

In conclusion, the results of the current study suggest that the overexpression of CXCR1/2 was associated with the malignant progression of gastric carcinoma, and simultaneously with the expression of certain indicators of phosphorylation, proliferation, growth, apoptosis, angiogenesis, invasion and metastasis. It is possible that CXCR1/2, interacting with their ligands, activate ERK1/2 and AKT phosphorylation, which in turn mediates the expression of indicators of proliferation, growth, apoptosis, angiogenesis, invasion and metastasis; and activation of the signaling pathway results in poor progression of gastric carcinoma (Fig. 3). Therefore, CXCR1/2 may be a useful predictive marker and promising therapeutic target in gastric carcinoma. Further research is required to confirm the relevance of CXCR1/2 expression to gastric carcinoma progression *in vitro* and *in vivo*.

## Figures and Tables

**Table I.** Patient data and tumor characteristics.

Factor	Value
No. of patients	69
Gender, n (%)	
Male	55 (73.5)
Female)	14 (26.5)
Age (years), median (range)	55 (31–79)
TNM stage, n (%)	
T stage	
T1	3 (4.3)
T2	13 (18.8)
T3	36 (52.2)
T4	17 (24.6)
N stage	
N0	22 (31.9)
N1	21 (30.4)
N2	12 (17.4)
N3	14 (20.3)
Overall stage	
I	8 (11.6)
II	33 (47.8)
III	27 (39.1)
IV	1 (1.4)
Differentiation	
Good	7 (10.1)
Moderate	24 (34.8)
Poor	38 (55.1)

**Table II.** Association between the expression of CXCR1/2 and clinicopathological factors of gastric carcinoma.

	Expression			
Characteristics	Negative (n=1)	Weak (n=45)	Strong (n=23)	P-value
Male:female	0:1	37:8	18:5	0.452
Age (years), mean ± SD	53.0±0	52.3±20.0	56.0±27.3	0.501
Cancer cell differentiation				0.357
Good	0	5	2	
Moderate	0	17	7	
Poor	1	23	14	
T stage				<0.001
T1	0	3	0	
T2	0	13	0	
T3	1	20	15	
T4	0	9	8	
N stage				<0.001
N0	1	20	1	
N1	0	17	4	
N2	0	7	5	
N3	0	1	13	
Overall stage				<0.001
I	0	8	0	
II	1	30	2	
III	0	6	21	
IV	0	1	0	

CXCR1/2, C-X-C chemokine receptor types 1/2.

**Table III.** Association between the expression of CXCR1/2 and indicators of proliferation, growth, apoptosis, angiogenesis, invasion and metastasis.

	CXCR1/2 expression			
Indicator expression	Negative (n=1)	Weak (n=45)	Strong (n=23)	P-value
Phosphorylation				
AKT				0.339
Negative	0	5	0	
Weak	1	25	13	
Strong	0	15	10	
pAKT				0.032
Negative	1	1	1	
Weak	0	15	6	
Strong	0	29	16	
ERK				0.725
Negative	0	0	0	
Weak	1	9	6	
Strong	0	36	17	
pERK				<0.001
Negative	0	2	0	
Weak	1	19	1	
Strong	0	24	22	
Proliferation and growth				
Ki-67				0.456
Negative	1	6	5	
Weak	0	28	11	
Strong	0	11	7	
Cyclin D1				0.049
Negative	0	8	2	
Weak	1	32	13	
Strong	0	5	8	
EGFR				0.013
Negative	1	14	3	
Weak	0	25	12	
Strong	0	6	8	
Apoptosis				
Bcl-2				0.003
Negative	0	4	0	
Weak	1	21	10	
Strong	0	20	13	
Bax				0.103
Negative	1	6	3	
Weak	0	34	15	
Strong	0	5	5	
Angiogenesis				
VEGF				0.678
Negative	0	2	1	
Weak	1	23	12	
Strong	0	20	10	
MVD				0.001
<20	1	31	9	
≥20	0	14	14	
Invasion and metastasis				
MMP-9				0.013
Negative	1	11	1	
Weak	0	29	16	
Strong	0	5	6	
MMP-2				0.027
Negative	1	14	6	
Weak	0	30	15	
Strong	0	1	2	
TIMP-2				0.843
Negative	0	24	11	
Weak	1	21	11	
Strong	0	0	1	
E-cadherin				0.414
Negative	0	12	4	
Weak	1	28	17	
Strong	0	5	2	

Tumor samples of patients were divided into negative, weak and positive groups of immunohistochemical expression. The tumor samples were divided into high and low MVD groups assessed with the mean microvessel density value of 20 as the cut-off value. CXCR1/2, C-X-C chemokine receptor types 1/2; MVD, microvessel density.

**Table IV.** Association between the expression of indicators of phosphorylation and those of proliferation, growth, apoptosis, angiogenesis, invasion and metastasis.

	P-value			
Indicator expression	AKT	pAKT	ERK	pERK
Proliferation and growth				
Ki-67	0.125	0.001	0.020	0.013
Cyclin D1	0.889	0.349	0.596	0.098
EGFR	0.296	0.029	0.370	0.002
Apoptosis				
Bcl-2	0.051	<0.001	0.003	<0.001
Bax	0.031	0.451	0.012	0.081
Angiogenesis				
VEGF	0.086	0.003	0.048	0.102
MVD	0.079	0.841	0.560	0.073
Invasion and metastasis				
MMP-9	0.427	0.161	0.275	0.003
MMP-2	0.572	0.041	0.086	0.010
TIMP-2	0.167	0.456	0.587	0.084
E-cadherin	0.014	0.202	0.110	0.391

**Table V.** Univariate and multivariate analyses of clinicopathological variables and the expression of CXCR1/2 with regard to TNM stage.

	Univariate analysis			Multivariate analysis		
Variables	OR	95% CI	P-value	OR	95% CI	P-value
Gender (male vs. female)	0.754	0.246–2.312	0.062	0.610	0.074–5.053	0.646
Age (<60 vs. ≥60 years)	1.022	0.405–2.581	0.963	0.806	0.167–3.900	0.789
Differentiation (poor vs. moderate, good)	0.570	0.229–1.422	0.228	0.549	0.108–2.804	0.471
CXCR1/2 (negative, weak vs. strong)	39.291	9.061–169.864	<0.001	204.793	14.850–2827.081	<0.001
pAKT (negative, weak vs. strong)	1.193	0.412–3.463	0.744	5.435	0.775–38.081	0.089
pERK (negative, weak vs. strong)	5.186	1.786–15.059	0.002	2.049	0.336–12.516	0.437
Ki-67 (negative, weak vs. strong)	1.111	0.400–3.089	0.840	0.849	0.082–8.837	0.891
Cyclin D1 (negative, weak vs. strong)	1.506	0.474–4.783	0.488	1.563	0.166–14.688	0.697
EGFR (negative, weak vs. strong)	1.091	0.357–3.330	0.879	0.149	0.013–1.766	0.131
Bcl-2 (negative, weak vs. strong)	1.560	0.631–3.857	0.336	3.436	0.481–24.582	0.219
Bax (negative, weak vs. strong)	1.127	0.315–4.039	0.854	0.677	0.071–6.449	0.734
VEGF (negative, weak vs. strong)	0.604	0.242–1.507	0.280	0.499	0.072–3.483	0.483
MVD (<20 vs. ≥20)	13.383	4.280–41.846	<0.001	28.905	4.092–204.384	0.001
MMP-9 (negative, weak vs. strong)	0.988	0.290–3.367	0.985	0.288	0.020–4.166	0.361
MMP-2 (negative, weak vs. strong)	0.419	0.045–3.881	0.443	0.260	0.003–26.050	0.566
E-cadherin (negative, weak vs. strong)	0.245	0.052–1.121	0.075	0.067	0.003–1.697	0.101

Patients were divided into two groups of age assessed with 60 years as the cut-off value. CXCR1/2, C-X-C chemokine receptor types 1/2; OR, odds ratio; MVD, microvessel density; 95% CI, 95% confidence interval.

**Table VI.** Uni- and multivariate analyses of clinicopathological variables and the expression of CXCR1/2 with regard to T stage.

	Univariate analysis			Multivariate analysis		
Variables	OR	95% CI	P-value	OR	95% CI	P-value
Differentiation (poor vs. moderate, good)	0.283	0.108–0.745	0.011	0.268	0.072–0.997	0.049
CXCR1/2 (negative, weak vs. strong)	4.039	1.448–11.257	0.008	8.101	1.861–35.269	0.005
pAKT (negative, weak vs. strong)	1.579	0.614–4.063	0.343	5.382	1.203–24.095	0.028
Ki-67 (negative, weak vs. strong)	0.396	0.140–1.123	0.081	0.149	0.026–0.843	0.031
EGFR (negative, weak vs. strong)	0.451	0.146–1.387	0.165	0.126	0.020–0.787	0.027
MVD (<20 vs. ≥20)	3.855	1.448–10.433	0.007	3.040	0.904–10.237	0.072

CXCR1/2, C-X-C chemokine receptor types 1/2; OR, odds ratio; 95% CI, 95% confidence interval; MVD, microvessel density.

**Table VII.** Uni- and multivariate analyses of clinicopathological variables and the expression of CXCR1/2 with regard to N stage.

	Univariate analysis			Multivariate analysis		
Variables	OR	95% CI	P-value	OR	95% CI	P-value
CXCR1/2 (negative, weak vs. strong)	23.571	7.121–77.945	<0.001	29.108	5.807–146.057	<0.001
pERK (negative, weak vs. strong)	5.155	1.910–13.902	0.001	5.523	1.283–23.807	0.022
EGFR (negative, weak vs. strong)	2.983	1.012–8.802	0.047	7.243	1.022–51.367	0.048
VEGF (negative, weak vs. strong)	0.610	0.257–1.449	0.262	0.106	0.022–0.514	0.005
MVD (<20 vs. ≥20)	5.686	2.201–14.673	<0.001	6.398	1.779–23.035	0.004

CXCR1/2, C-X-C chemokine receptor types 1/2; OR, odds ratio; 95% CI, 95% confidence interval; MVD, microvessel density.
